# Dietary Risk Factors for Cardiovascular Disease among Low-Income Haitian Adults: Findings from a Population-Based Cohort

**DOI:** 10.3390/nu14040787

**Published:** 2022-02-13

**Authors:** Adrienne Clermont, Rodney Sufra, Jean Lookens Pierre, Michelle Nour Mourra, Elizabeth L. Fox, Vanessa Rouzier, Eliezer Dade, Stephano St-Preux, Joseph Inddy, Hilaire Erline, Fleurijean Pierre Obed, Lily D. Yan, Miranda Metz, Myung Hee Lee, Daniel W. Fitzgerald, Marie Marcelle Deschamps, Jean W. Pape, Margaret L. McNairy

**Affiliations:** 1 Center for Global Health, Weill Cornell Medicine, 402 East 67th Street, New York, NY 10065, USA; liy9032@med.cornell.edu (L.D.Y.); mim4018@med.cornell.edu (M.M.); myl2003@med.cornell.edu (M.H.L.); dwf2001@med.cornell.edu (D.W.F.); jwp2001@med.cornell.edu (J.W.P.); mam9365@med.cornell.edu (M.L.M.); 2 MD Program, Weill Cornell Medicine, 1300 York Avenue, New York, NY 10065, USA; 3 Haitian Group for the Study of Kaposi’s Sarcoma and Opportunistic Infections (GHESKIO), 33 Boulevard Harry Truman, Port-au-Prince 6110, Haiti; rsufra@gheskio.org (R.S.); lookensdocp@gmail.com (J.L.P.); vrouzier@gheskio.org (V.R.); eliezerdade@yahoo.fr (E.D.); stpreuxstephano@gheskio.org (S.S.-P.); inddy.joseph@gmail.com (J.I.); erlinehilaire92@gmail.com (H.E.); fpobed@yahoo.com (F.P.O.); mariehd@gheskio.org (M.M.D.); 4 Department of Public and Ecosystem Health, Cornell University, S2005 Schurman Hall, Ithaca, NY 14853, USA; mhm265@cornell.edu (M.N.M.); elf23@cornell.edu (E.L.F.)

**Keywords:** nutrition, Haiti, cardiovascular disease, noncommunicable disease, dietary habits

## Abstract

Poor diets are responsible for a large burden of noncommunicable disease (NCD). The prevalence of modifiable dietary risk factors is rising in lower-income countries such as Haiti, along with increasing urbanization and shifts to diets high in sugar, salt, and fat. We describe self-reported dietary patterns (intake of fruits, vegetables, fried food, sugar-sweetened beverages, and added salt and oil) among a population-based cohort of low-income adults in Port-au-Prince and assess for associated sociodemographic factors (age, sex, income, education, body mass index). Among 2989 participants, the median age was 40 years, and 58.0% were women. Less than 1% met the World Health Organization recommendation of at least five servings/day of fruits and vegetables. Participants consumed fried food on average 1.6 days/week and sugar-sweetened beverages on average 4.7 days/week; young males of low socioeconomic status were the most likely to consume these dietary risk factors. The vast majority of participants reported usually or often consuming salt (87.1%) and oil (86.5%) added to their meals eaten at home. Our findings underscore the need for public health campaigns, particularly those targeting young males and household cooks preparing family meals at home, to improve dietary patterns in Haiti in order to address the growing NCD burden.

## 1. Introduction

Poor diets are responsible for one in five deaths globally, more than any other behavioral risk factor [1]. Historically, in low- and middle-income countries (LMICs), diet-related risks have focused on undernutrition (e.g., inadequate calories and micronutrient deficiencies) [2]. However, the burden of malnutrition in LMICs is transitioning with changing food environments, increasing urbanization, and shifts to diets high in saturated and trans fats, added sugars, and salt [3]. Many countries now face multiple burdens of malnutrition, with persistent undernutrition existing alongside increasing overweight/obesity and resultant noncommunicable diseases (NCDs) [3]. 

Only 67% of Haitian households have a nutritionally diverse diet, and nearly 51% of households suffer from food insecurity, according to national survey data from 2019 [4]. At the same time, similar to global trends, there is increasing consumption of foods that are high in added sugar, salt, and fat, particularly in urban areas such as Port-au-Prince [3,5]. From 2012 to 2016, the proportion of the population that is overweight or obese (body mass index [BMI] ≥ 25.0 kg/m^2^) increased from 25% to 32% [6]. In urban regions of Haiti, 39% of men and 46% of women were found to have elevated blood pressure [6]. Cardiovascular disease (CVD) is now the leading cause of death among Haitian adults [7], and modeling studies estimate that over 45% of CVD deaths and disability in Haiti can be attributed to dietary risk factors [1]. 

In order to better target future interventions to address the growing burden of disease in this setting, there is a need to more explicitly and systematically examine dietary risk factors associated with NCDs. To address the gap in the understanding of these patterns in urban Haiti, we describe self-reported dietary data—including fruit and vegetable intake and consumption of foods high in added sugar, salt, and fat—among a population-based cohort in Port-au-Prince. We also assess for sociodemographic factors associated with these dietary patterns. 

## 2. Materials and Methods

### 2.1. Study Setting and Population 

This paper reports cross-sectional data from 2989 adult participants enrolled in the Haiti CVD Cohort study (Clinicaltrials.gov registration number NCT03892265) in Port-au-Prince, the capital of Haiti [8]. The Haiti CVD Cohort study aims to estimate the prevalence and incidence of CVD risk factors and diseases. The study recruited adult participants in the community via multistage random sampling using GPS waypoints based on national census blocks, as has been previously described [8]. The number of waypoints was proportional to the estimated size of the population in each block, and members of each selected household were randomly selected and invited to enroll in the study. Participants completed enrollment procedures at GHESKIO (Groupe Haitien d’Etude du Sarcome de Kaposi et des Infections Opportunistes), a clinic and training center founded in 1982 and located in downtown Port-au-Prince. All participants were aged 18 years or greater and were enrolled in the study between March 2019 and August 2021.

### 2.2. Measurements

All study data were collected by GHESKIO research staff and community health workers who have completed Good Clinical Practice research training as well as training on the study-specific data collection instruments. Self-reported dietary data were collected using a questionnaire adapted from the World Health Organization STEPwise Approach to NonCommunicable Disease Surveillance (WHO STEPS) methodology, which was developed to create standardized, longitudinal data on modifiable NCD risk factors at the national level, allowing for comparison between countries and over time [9,10]. Since its launch in 2002, WHO STEPS surveys have been conducted in 134 countries and territories around the world. The survey includes self-reported data on dietary risk factors (e.g., tobacco use, alcohol consumption, fruit and vegetable intake, salt use, and physical activity).

At study enrollment, Haiti CVD Cohort participants were asked to answer a series of 12 questions regarding their dietary practices during a typical week (Table 1). Of these questions, six (those regarding fruits, vegetables, and salt) were from the WHO STEPS methodology [11]. Participants also answered six diet questions regarding meal sources, use of cooking oil, and intake of sugar-sweetened beverages, which were included based on formative research regarding local dietary patterns. The questionnaire was developed in English and translated into Haitian Creole by bilingual staff. Reference cards showing photos of relevant food types and portion sizes were used to assist respondents in reporting their dietary consumption.

Sociodemographic data were also collected for all participants, including age, sex, education level, height and weight (used to calculate BMI), and income level. Education was categorized as having completed no education, primary, secondary, or higher than secondary education. Height and weight were measured during a clinical exam with trained study nurses and physicians. Income was measured using categorical answer choices in Haitian gourdes (HTG) and then converted to U.S. dollars (USD) for comparability (1 USD = roughly HTG 90 during the study period). For the purposes of this analysis, income was dichotomized into two categories: lower income (all income categories from no income up to HTG 1000 [11 USD] per day) and higher income (greater than HTG 1000 [11 USD] per day).

### 2.3. Dietary Outcomes

Seven dietary outcomes are explored in this analysis, with detailed definitions shown in Table 1. Fruit consumption and vegetable consumption were collected as the average number of days consumed per week and average number of servings per day on a day when they are consumed. These values were used to calculate a continuous variable for the average number of servings per day of fruits and vegetables. Fried food consumption and sugar-sweetened beverage consumption were collected as a numerical variable of the average number of days per week consuming these categories. Eating at a street vendor and eating in a restaurant/cafeteria were collected as a numerical variable of the average number of days per week eating at one of these locations. Home salt consumption was calculated as a dichotomous variable with higher consumption defined as answering “usually/often” to one or both of questions 9 and 10 (see Table 1), and lower consumption was defined as answering “sometimes” or “rarely/never” to both questions. Home cooking oil consumption was calculated similarly, using the responses to questions 11 and 12 (see Table 1).

### 2.4. Statistical Analysis

The full dataset for the Haiti CVD Cohort includes 3005 participants. For this analysis, 16 participants were excluded due to missing data. Thus, the analysis described here includes 2989 participants with complete enrollment data.

Data were analyzed using Stata version 13 (College Station, TX, USA: StataCorp LP). Following exploratory data analysis, univariate regression analyses were performed to explore the associations between sociodemographic factors and the dietary outcomes of interest. Linear regression was used for the continuous outcome, Poisson regression for the discrete numerical outcomes, and logistic regression was used for the dichotomous outcomes. The nonnormally distributed continuous outcome (fruit and vegetable consumption) was log-transformed prior to regression analysis. For each outcome of interest, we used backward selection from the full model until we arrived at the final multivariable regression model with all remaining factors significant with *p*-values of <0.05.

### 2.5. Ethics Approval

This study was approved by the institutional review boards at Weill Cornell Medicine and GHESKIO in Haiti (IRB #1803019037). Prior to study implementation, meetings were held with community, school, and religious leaders, as well as GHESKIO’s Community Advisory Board, to answer questions regarding the study. Written consent was obtained for all participants prior to enrollment.

## 3. Results

The sociodemographic characteristics of the study population are described in Table 2. The study population was predominantly female (58.0%) and had a wide age range from 18 to 93 years. The majority of participants (64.2%) had completed secondary education or higher. About half of the participants (46.5%) had a normal BMI, with a small minority (6.8%) in the underweight category and 46.7% in the overweight and obese categories. The vast majority of participants (82.3%) were in the lower-income category, including 67.3% of participants who reported making no income.

### 3.1. Fruit and Vegetable Consumption

Participants reported eating fruit on average 2.5 days per week (standard deviation (SD): 2.4) and an average of 0.9 servings of fruit per day (SD: 1.0), with 805 participants (26.9%) reporting never eating fruit in a typical week. Participants reported eating vegetables on average 2.0 days per week (SD: 1.8) and an average of 1.0 servings of vegetables per day (SD: 0.9), with 615 participants (20.6%) reporting never eating vegetables in a typical week.

**Figure 1:**
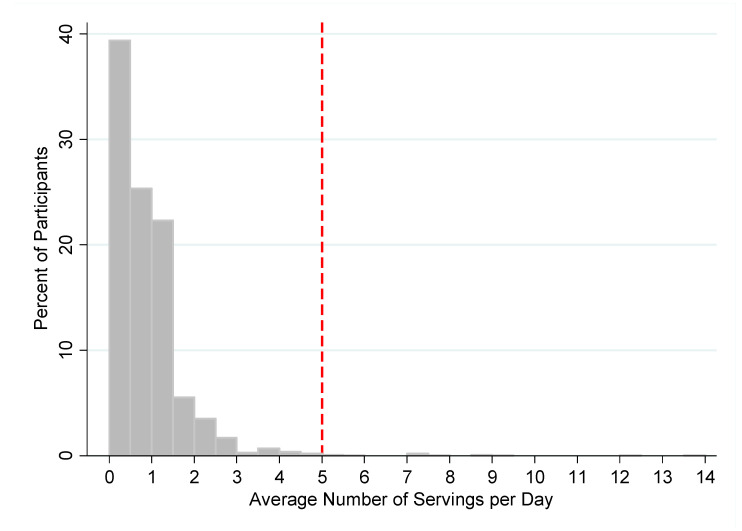
Average daily consumption of fruits and vegetables among study participants. Note: red dotted line indicates World Health Organization recommendation for daily servings of fruits and vegetables.

Combining the consumption of both fruits and vegetables, participants reported eating an average of 0.8 servings per day (SD: 0.9), including 291 participants (9.7%) who reported eating zero servings of fruits and vegetables in a typical week. Only 19 participants (0.6%) reached the WHO recommendation [12] of five servings per day of fruits and vegetables (Figure 1). Using more moderate cutoffs, 1055 participants (35.3%) ate one or more servings/day of fruits and vegetables, and 66 respondents (2.2%) ate three or more servings/day.

Because the data were not normally distributed, fruit and vegetable consumption was log-transformed prior to performing regression analysis. To interpret the regression results in the original scale, we exponentiated the coefficients and have presented the percent change in fruit and vegetable consumption, relative to the reference category, in Table 3. In the multivariable regression analysis, a higher income (relative to a lower income) and a BMI ≥ 30 kg/m^2^ (relative to a normal BMI) were associated with higher consumption of fruits and vegetables.

### 3.2. Fried Food Consumption

**Figure 2:**
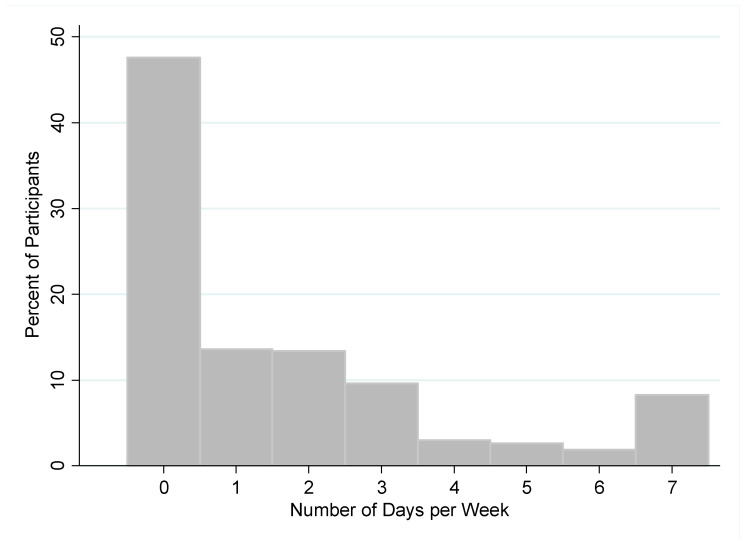
Weekly consumption of fried food among study participants.

Participants reported eating fried food on average 1.6 days per week (SD: 2.2) (Figure 2). Nearly half of the participants (1422 participants; 47.6%) reported never eating fried food in a typical week, while 247 participants (8.3%) reported eating fried food every day in a typical week.

In the multivariable regression analysis (Table 4), each increasing age group was associated with lower consumption of fried food (relative to the youngest age group). Participants under age 30 reported eating fried food on average 2.4 days per week, while participants over age 60 reported eating fried food on average 1.1 days per week.

### 3.3. Sugar-Sweetened Beverage Consumption

**Figure 3:**
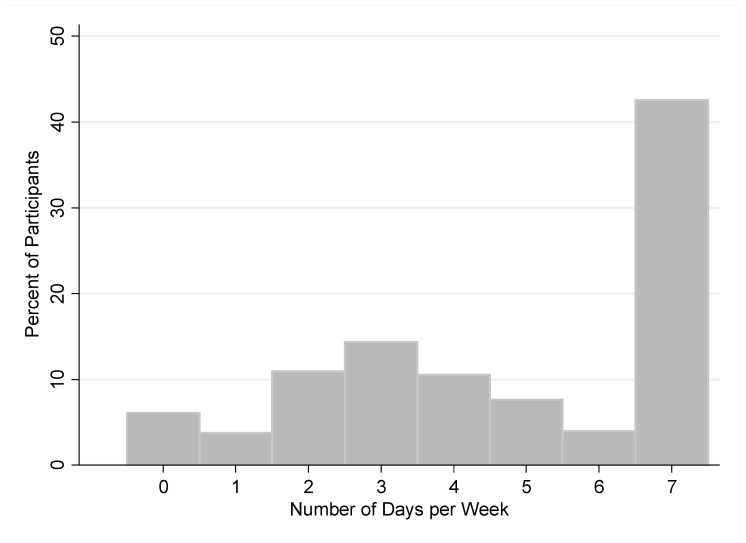
Weekly consumption of sugar-sweetened beverages among study participants.

Participants reported drinking sugar-sweetened beverages on average 4.7 days per week (SD: 2.3) (Figure 3). Only 183 participants (6.1%) reported never drinking sugar-sweetened beverages in a typical week, while 1272 respondents (42.6%) reported drinking them every day in a typical week.

In the multivariable regression analysis (Table 5), age groups 50–59 and ≥60 (relative to the youngest age group) were associated with lower sweetened beverage consumption, and secondary education (relative to no education) was associated with higher sugar-sweetened beverage consumption. Participants under age 30 reported drinking sugar-sweetened beverages on average 5.1 days per week, while participants over age 60 reported drinking them on average 4.0 days per week.

### 3.4. Eating Outside the Home

**Figure 4:**
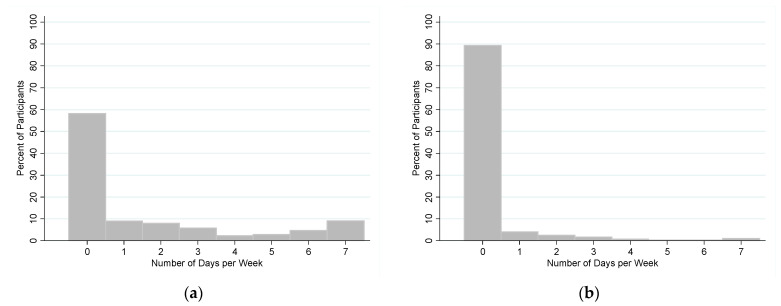
(**a**) Frequency of eating meals from a street vendor and (**b**) frequency of eating meals from a restaurant or cafeteria.

Participants reported eating a meal prepared by a street vendor on average 1.6 days per week (SD: 2.4) (Figure 4a). The majority of participants (1738 people; 58.1%) reported that they never ate at a street vendor in a typical week. 

In the multivariable regression analysis (Table 6), an age ≥ 60 (relative to the youngest age group), female sex (relative to males), having complemented greater than secondary education (relative to no education), and a BMI ≥ 30 kg/m^2^ (relative to normal BMI) were all associated with a lower number of days eating a meal from a street vendor. Of note, older age and female sex were strongly associated with less education; due to this association between covariates, the regression coefficients for education, while accounting for age and sex, differ from the coefficients in the univariate analysis. Male participants reported eating at a street vendor on average 2.2 days per week, while female participants reported eating at a street vendor on average 1.1 days per week.

Participants reported eating at a restaurant or cafeteria on average 0.3 days per week (SD: 1.0) (Figure 4b). An overwhelming majority of participants (2668 people; 89.3%) reported that they never ate at a restaurant or cafeteria in a typical week. Due to the homogeneity of these results, a regression analysis was not performed.

### 3.5. Salt and Oil Use in the Home

**Figure 5:**
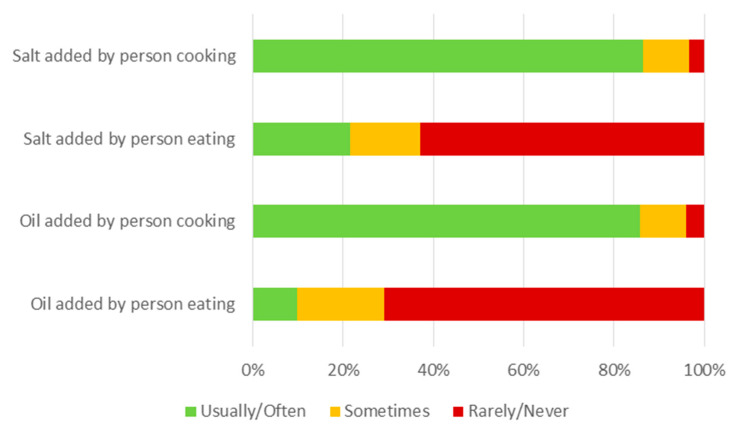
Frequency of adding salt and oil to food by participants and household cooks.

Participant responses to the questions regarding the use of salt (including salty sauces and seasonings) and oils (including butter and margarine) in home cooking and during meal consumption are shown in Figure 5. Regarding salt products, 2603 participants (87.1%) answered “usually/often” to at least one of the questions about the addition of salt to their food by either the person cooking or the person eating and were thus classified in the “higher use” category for salt. Only 91 respondents (3.0%) answered “rarely/never” to both questions about the addition of salt to food during cooking or consumption. 

Regarding cooking oils, 2585 respondents (86.5%) answered “usually/often” to at least one of the questions about the addition of oils to their food by either the person cooking or the person eating and were thus classified in the “higher use” category for oils. Only 102 respondents (3.4%) answered “rarely/never” to both questions about the addition of oils to food during cooking or consumption.

In the multivariable regression analyses for both salt use (Table 7) and oil use (Table 8), age groups 40–49, 50–59, and ≥60 (relative to the youngest age group); female sex (relative to males); and a higher income (relative to a lower income) were all associated with a greater likelihood of being in the “lower use” category.

## 4. Discussion

In line with global trends, our analysis reports a high prevalence of dietary risk factors for CVD among low-income urban Haitians, including low intake of fruits and vegetables, frequent consumption of fried foods and sugar-sweetened beverages, and the frequent use of salt and oil in home cooking. These behaviors are often concurrent, with the most at-risk populations exposed to multiple dietary risk factors (Supplementary Table S1).

### 4.1. Fruit and Vegetable Consumption

High consumption of fruits and vegetables is a protective dietary factor for reduced CVD risk. The WHO recommends consuming five servings a day of fruit and vegetables, with a mortality benefit supported by large-scale observational studies in the United States [13]. An overwhelming 99.4% of the Haiti CVD Cohort participants failed to meet this target; only 35.3% of participants reported that they eat an average of one serving of fruits and vegetables a day. These findings align with our prior study in four slum neighborhoods of Port-au-Prince in 2016, which reported that fruits and vegetables are only consumed an average of 1.6 days per week [5]. WHO STEPS surveys in other countries in the Caribbean region and other countries with a similar gross domestic product to Haiti [9] (Supplementary Table S2) as well as other surveys in LMICs [14] have shown comparable levels of low fruit and vegetable consumption. Consumption is low even in high-income countries, with more than 90% of adults in the United States failing to meet the “five a day” guideline in 2015 [15].

A higher income was associated with increased intake of fruits and vegetables in this study, suggesting that financial means may play a determining role in consumption patterns. Socioeconomic factors have been shown to greatly influence dietary choices and patterns. According to the Food and Agriculture Organization, 87% of people living in low-income countries cannot afford to consume a healthy diet [16]. Among the study population, a BMI ≥ 30 kg/m^2^ (relative to a lower BMI) was associated with increased fruit and vegetable consumption. This may be due to greater dietary intake overall for these participants, but the reasons for this association warrant further research. 

Low consumption of fruits and vegetables can be attributed to high relative costs, lack of availability, and concerns about perishability [16,17,18,19]. In a recent national study, only 40.9% of households in Haiti had electricity [6], which would impact residents’ ability to store and refrigerate highly perishable foods such as produce and animal-source products. Political instability leading to violent demonstrations, fuel shortages, and rapid inflation has further affected food security in recent years; the average cost of a food basket is estimated to have risen 30% from 2019 to 2020 [20]. The Haitian diet is often dependent on nonperishable, calorie-rich starches, including rice, plantains, potatoes, cassava, and corn [21], and vegetables are typically cooked into stews or sauces, which impacts portion size and nutritional benefits [22]. Nonperishable, packaged foods are widely available and affordable in Haiti through the global supply chain, and although many consumers are aware that they may be less healthy, aspirational desire and low prices influence preferences for these foods in lieu of fresh fruits and vegetables [23,24].

### 4.2. Dietary Risk Factors

Fried food consumption among the study population was variable, with around half of the participants (47.6%) reporting never eating fried food, while 8.3% reported eating fried food every day in a typical week. These values are lower than estimates from the 2016 Port-au-Prince study, which indicated that 68.9% of participants consumed at least one fried meal per day [5]. It is possible that our results underreport the true prevalence, as consumers may not know how their food is prepared if they did not cook it themselves (whether consuming food at home or from a street vendor). Additionally, our study found that age was inversely associated with fried food consumption, suggesting that young people are the most likely to eat these unhealthy foods. This aligns with other studies in Haiti and other places around the world which document higher consumption of fried foods and associated fats (e.g., trans fats) among adolescents and young adults, particularly those in urban areas. For instance, in one study, rural-to-urban migrant youth in Haiti reported higher consumption of fried snacks and sugar-sweetened beverages compared to their peers in rural areas [25]. Convenience, peer influence, and low cost are often associated with young adults’ consumption of these foods [25,26].

Sugar-sweetened beverage consumption was extremely high, with 93.9% of participants reporting that they drink them at least once in a typical week, and 42.6% of participants reporting drinking them every day. This aligns with the high reported consumption of sugar-sweetened beverages in Caribbean countries [27]. These beverages typically retail for HTG 50 (approximately USD 0.55) and are widely available for purchase from sidewalk vendors throughout Port-au-Prince. There are numerous reasons why consumption of sugar-sweetened beverages is high, including wide availability, marketing and advertising, taste preferences, and their low cost [3,27,28]. Water safety concerns are also likely relevant to consumption of sugar-sweetened beverages in Port-au-Prince, as has been reported in other settings [29,30,31]. For instance, a study in Canaan, a neighborhood in Port-au-Prince, showed that on average 16% of the head of household’s income was spent on purchasing treated water for daily consumption, due to the lack of potable water sources in this neighborhood [32]. Similar to fried foods, younger age was associated with significantly higher consumption of sugar-sweetened beverages.

Among the study population, it appears that the majority of meals are cooked in the home, with 58.1% of participants reporting that they never ate a meal from a street vendor and 89.3% of participants reporting that they never ate a meal from a restaurant or a cafeteria in a typical week. Younger, less-educated males were the group most likely to purchase meals from a street vendor. This finding aligns with growing evidence of increasing street food consumption globally [33] and may be because young men are most likely to work away from home. For example, in a study in South Africa, males consumed a higher percentage of street food and fast food compared to women [34]. Convenience and cost-efficiency were cited as contributing factors to these trends.

For those meals cooked at home, the addition of salt and oils is highly prevalent. Consumption of butter and margarine has been associated with increased CVD mortality [35], and research suggests that repeatedly heating and reusing cooking oil—a practice prevalent in low-income settings such as Haiti—can also have harmful health effects [36]. However, it is important to note that participants were not asked to differentiate between potentially healthier items, such as vegetable oil, and more unhealthy items, such as animal-derived saturated fats, so it is difficult to assess the full health impact of this category. Similarly, higher salt intake is associated with increased hypertension and CVD mortality [37]. In 2013, the World Health Assembly adopted the global target of a 30% reduction in the mean population intake of salt for noncommunicable disease control [38]. However, previous research in Haiti suggests that average salt consumption is well above the WHO-recommended maximum of 5 g per day [39,40,41] and is culturally important for a number of reasons, including taste preferences and food preservation [42].

### 4.3. Future Research and Interventions

Increased consumption of sugar, salt, and fats contributes to an increased CVD risk [1]. Our findings suggest several areas of exploration for future research and public health interventions targeting dietary drivers of CVD. 

First, our findings suggest that public health and nutritional programs, to increase consumption of fruits and vegetables in Haiti, will need to address barriers of both access and affordability. Further formative research is necessary to understand which factors are the strongest barriers against fruit and vegetable consumption and to map the availability of fresh foods (or conversely, the presence of “food deserts” or “food swamps”) in low-income slum areas of Port-au-Prince.

Second, our study indicates that young people, particularly young males of low socioeconomic status, were the subgroup most likely to consume dietary risk factors of CVD such as fried foods and sugar-sweetened beverages. This dietary pattern may contribute to the early onset of hypertension seen in Haitians as compared to African Americans in the United States [5]. A greater understanding of the preferences and motivations of this subgroup will allow for better targeting of public health campaigns in the future. Other harmful dietary factors, such as sweet and salty prepackaged snack foods, should be investigated as well. Intervening with the youth population could be an important avenue for primary prevention, prior to the onset of CVD. 

Third, the majority of meals were eaten at home (rather than purchased from street vendors or restaurants), and the use of salty seasonings and oils by home cooks was high. Health and nutrition awareness campaigns aimed at household cooks who prepare meals for their families could be an effective way to decrease salt consumption in line with global guidelines. A better understanding of the specific sources of salt and fat in Haitian home cooking, as well as the knowledge and attitudes of household cooks, will help to inform future public health initiatives.

Once these three areas are better understood, the Haitian government and other public health actors should take action to improve the dietary habits of low-income Haitians. This might include health education campaigns, targeted taxes on specific harmful food categories, or vouchers for the purchase of fresh produce. However, any future interventions must bear in mind the extreme financial constraints faced by the low-income population in Haiti. Food is the greatest expense for Haitian households, accounting for 70% of spending on average [4], resulting in highly price-sensitive consumers [24]. Efforts to promote a healthier and more nutritious diet must take cost factors into account, as well as taste and cultural preferences. 

### 4.4. Strengths and Limitations

Strengths of this study include the use of a population-based cohort and diet measures comparable to other international studies. Nevertheless, self-reported dietary data have been shown in other settings to be vulnerable to recall bias and social desirability bias. In high-income settings, people tend to over-report consumption of fruits and vegetables and of foods that are perceived to be healthier and to under-report their consumption of less healthy foods [43,44]. Although there are limited studies examining measurement errors in LMICs [45], some studies in these settings have shown trends of under-reporting of food intake among men and women of low socioeconomic status, especially in older age groups [46,47,48]. In addition, although locally relevant examples of food categories were provided, participants may have left out items that were not listed as examples or shown on the photo cards. Finally, our cohort is exclusively from low-income urban areas of Port-au-Prince, so our findings cannot be extrapolated to Haiti as a whole and are not directly comparable to nationally representative surveys. 

## 5. Conclusions

LMICs such as Haiti face an increasing prevalence of dietary risk factors for CVD. Our study among low-income urban Haitians shows a strikingly low consumption of protective foods such as fruits and vegetables and high consumption of dietary risk factors such as fried foods, sugar-sweetened beverages, and added salt and oils. Key populations related to these risk factors are young, low-income males and household cooks preparing meals at home. Further research is necessary to understand knowledge and attitudes around diet and disease risk in Haiti, as well as the most effective way to shape resulting interventions.

## Figures and Tables

**Table 1:** Dietary questions from Haiti CVD Cohort study enrollment.

Questions	Definitions and Locally Relevant Examples	Outcomes
1. In a typical week, on how many days do you eat fruit?	Avocado, mango	Average number of servings of fruits and vegetables per day = [(number of days per week eating fruit) × (number of daily servings of fruit) + (number of days per week eating vegetables) × (number of daily servings of vegetables)] ÷ 7
2. How many servings of fruit do you eat on a typical day?	1 serving = ½ cup or 1 medium fruit (can be raw or cooked, but fruit juice and canned fruits are not included)	
3. In a typical week, on how many days do you eat vegetables?	Potatoes, pumpkin, carrots, spinach	
4. How many servings of vegetables do you eat on a typical day?	1 serving = ½ cup (or 1 cup for leafy raw vegetables)	
5. In a typical week, how many days do you eat a meal that contains fried foods?	Fried plantains, tubers, or pork (includes foods from any source)	Average number of days per week eating fried foods
6. In a typical week, how often do you drink soda or sweetened fruit drinks, sports or energy drinks?	Tampico-brand juice, Toro-brand energy drink	Average number of days per week drinking sugar-sweetened beverages
7. In a typical week, how many days do you eat a meal prepared by a street vendor?		Average number of days per week eating a meal from a street vendor
8. In a typical week, how many days do you eat a meal prepared by a restaurant or cafeteria?		Average number of days per week eating a meal from a restaurant/cafeteria
9. How often is salt or salt-containing seasoning used by the person cooking or preparing foods at home?	Maggi-brand bouillon cubes, garlic salt, onion salt, soy sauce, fish sauce (includes only meals cooked at home)	Higher intake category = proportion of participants answering “usually/often” to one or both of questions 9 and 10
10. How often do you add salt or salt-containing seasoning to your food before you eat it or when you are eating it?		
11. How often is oil, butter, or margarine used by the person cooking or preparing foods at home?	Gourmet-brand oil, Marianne-brand margarine, Ti-Malice-brand butter (includes only meals cooked at home)	Higher intake category = proportion of participants answering “usually/often” to one or both of questions 11 and 12
12. How often do you add oil, butter, or margarine to your food before you eat it or when you are eating it?		

**Table 2:** Sociodemographic characteristics of study participants.

	Number (%)
Total Participants	2989 (100%)
**Sex**	
Male	1255 (42.0%)
Female	1734 (58.0%)
**Age**	
18–29 years	881 (29.5%)
30–39 years	565 (18.9%)
40–49 years	531 (17.8%)
50–59 years	498 (16.7%)
≥60 years	514 (17.2%)
**Education**	
None	427 (14.3%)
Primary	646 (21.6%)
Secondary	1475 (49.4%)
Higher than secondary	441 (14.8%)
**BMI**	
Underweight (<18.5 kg/m^2^)	203 (6.8%)
Normal (18.5–24.9 kg/m^2^)	1391 (46.5%)
Overweight (25–29.9 kg/m^2^)	830 (27.8%)
Obese (≥30.0 kg/m^2^)	565 (18.9%)
**Income**	
Lower income	2459 (82.3%)
Higher income	530 (17.7%)

Percentages may not sum to 100% due to rounding. BMI = body mass index.

**Table 3:** Associations between sociodemographic characteristics and servings per week of fruit and vegetable consumption among the study population.

	Univariate Analysis		Multivariable Analysis	
Variable	Percent Change ** [95% CI]	*p*-Value	Percent Change ** [95% CI]	*p*-Value
**Age (ref = 18–29 years)**				
30–39 years	7% [3%, 12%]	<0.01 *		
40–49 years	7% [3%, 12%]	<0.01 *		
50–59 years	4% [−1%, 9%]	0.09		
≥60 years	0% [−4%, 5%]	0.93		
**Sex (ref = Male)**				
Female	0% [−3%, 3%]	0.90		
**Education (ref = None)**				
Primary	0% [−5%, 5%]	0.85		
Secondary	2% [−2%, 7%]	0.29		
Greater than secondary	4% [−2%, 9%]	0.17		
**BMI (ref = Normal [18.5–24.9 kg/m^2^])**				
Underweight (<18.5 kg/m^2^)	3% [−3%, 9%]	0.39	4% [−2%, 10%]	0.22
Overweight (25–29.9 kg/m^2^)	5% [1%, 8%]	0.01 *	3% [0%, 7%]	0.06
Obese (≥30.0 kg/m^2^)	9% [5%, 14%]	<0.01 *	7% [3%, 11%]	<0.01 *
**Income (ref = Lower income)**				
Higher income	22% [17%, 26%]	<0.01 *	21% [17%, 26%]	<0.01 *

* Statistically significant, *p* < 0.05. ** Linear regression performed on log-transformed outcome. For interpretation on the original scale, regression coefficients were converted to percent change in servings per week of fruit and vegetable consumption: (2^coefficient^ − 1) × 100. BMI = body mass index, CI = confidence interval.

**Table 4:** Associations between sociodemographic characteristics and number of days per week consuming fried food among the study population.

	Univariate Analysis		Multivariable Analysis	
Variable	Coefficient [95% CI]	*p*-Value	Coefficient [95% CI]	*p*-Value
**Age (ref = 18–29 years)**				
30–39 years	−0.31 [−0.44, −0.19]	<0.01 *	−0.31 [−0.44, −0.19]	<0.01 *
40–49 years	−0.56 [−0.70, −0.42]	<0.01 *	−0.56 [−0.70, −0.42]	<0.01 *
50–59 years	−0.68 [−0.83, −0.52]	<0.01 *	−0.68 [−0.83, −0.52]	<0.01 *
≥60 years	−0.80 [−0.95, −0.65]	<0.01 *	−0.80 [−0.95, −0.65]	<0.01 *
**Sex (ref = Male)**				
Female	−0.11 [−0.20, −0.01]	0.03 *		
**Education (ref = None)**				
Primary	0.24 [0.06, 0.42]	0.01 *		
Secondary	0.53 [0.38, 0.69]	<0.01 *		
Greater than secondary	0.64 [0.46, 0.82]	<0.01 *		
**BMI (ref = Normal [18.5–24.9 kg/m^2^])**				
Underweight (<18.5 kg/m^2^)	0.07 [−0.10, 0.25]	0.41		
Overweight (25–29.9 kg/m^2^)	−0.07 [−0.18, 0.05]	0.25		
Obese (≥30.0 kg/m^2^)	−0.27 [−0.41, −0.13]	<0.01 *		
**Income (ref = Lower income)**				
Higher income	−0.12 [−0.24, 0.00]	0.06		

* Statistically significant, *p* < 0.05; BMI = body mass index, CI = confidence interval.

**Table 5:** Associations between sociodemographic characteristics and number of days per week consuming sugar-sweetened beverages among the study population.

	Univariate Analysis		Multivariable Analysis	
Variable	Coefficient [95% CI]	*p*-Value	Coefficient [95% CI]	*p*-Value
**Age (ref = 18–29 years)**				
30–39 years	0.02 [−0.02, 0.07]	0.28	0.03 [−0.02, 0.08]	0.19
40–49 years	−0.03 [−0.08, 0.02]	0.21	−0.02 [−0.07, 0.03]	0.42
50–59 years	−0.17 [−0.23, −0.12]	<0.01 *	−0.15 [−0.21, −0.08]	<0.01 *
≥60 years	−0.23 [−0.29, −0.17]	<0.01 *	−0.19 [−0.27, −0.12]	<0.01 *
**Sex (ref = Male)**				
Female	−0.02 [−0.05, 0.02]	0.36		
**Education (ref = None)**				
Primary	0.08 [0.01, 0.15]	0.02 *	0.05 [−0.02, 0.12]	0.16
Secondary	0.20 [0.14, 0.26]	<0.01 *	0.08 [0.01, 0.15]	0.02 *
Greater than secondary	0.20 [0.13, 0.27]	<0.01 *	0.06 [−0.02, 0.14]	0.13
**BMI (ref = Normal [18.5–24.9 kg/m^2^])**				
Underweight (<18.5 kg/m^2^)	−0.03 [−0.10, 0.04]	0.37		
Overweight (25–29.9 kg/m^2^)	−0.01 [−0.05, 0.04]	0.76		
Obese (≥30.0 kg/m^2^)	0.01 [−0.03, 0.06]	0.55		
**Income (ref = Lower income)**				
Higher income	0.02 [−0.03, 0.06]	0.41		

* Statistically significant, *p* < 0.05; BMI = body mass index, CI = confidence interval.

**Table 6:** Associations between sociodemographic characteristics and number of days per week eating meals at a street vendor among the study population.

	Univariate Analysis		Multivariable Analysis	
Variable	Coefficient [95% CI]	*p*-Value	Coefficient [95% CI]	*p*-Value
**Age (ref = 18–29 years)**				
30–39 years	0.06 [−0.10, 0.21]	0.47	0.11 [−0.04, 0.26]	0.14
40–49 years	0.11 [−0.04, 0.26]	0.17	0.13 [−0.02, 0.29]	0.10
50–59 years	−0.12 [−0.30, 0.05]	0.16	−0.09 [−0.28, 0.09]	0.33
≥60 years	−0.32 [−0.50, −0.14]	<0.01 *	−0.35 [−0.55, −0.14]	<0.01 *
**Sex (ref = Male)**				
Female	−0.72 [−0.82, −0.61]	<0.01 *	−0.79 [−0.91, −0.67]	<0.01 *
**Education (ref = None)**				
Primary	0.09 [−0.12, 0.30]	0.40	−0.11 [−0.32, 0.09]	0.29
Secondary	0.35 [0.17, 0.53]	<0.01 *	−0.04 [−0.25, 0.16]	0.69
Greater than secondary	0.18 [−0.04, 0.40]	0.11	−0.28 [−0.53, −0.04]	0.02 *
**BMI (ref = Normal [18.5–24.9 kg/m^2^])**				
Underweight (<18.5 kg/m^2^)	−0.14 [−0.36, 0.08]	0.21	0.13 [−0.08, 0.35]	0.23
Overweight (25–29.9 kg/m^2^)	−0.18 [−0.31, −0.05]	<0.01 *	0.14 [−0.09, 0.37]	0.24
Obese (≥30.0 kg/m^2^)	−0.22 [−0.37, −0.07]	<0.01 *	0.28 [0.03, 0.53]	0.03 *
**Income (ref = Lower income)**				
Higher income	0.09 [−0.04, 0.22]	0.18		

* Statistically significant, *p* < 0.05; BMI = body mass index, CI = confidence interval.

**Table 7:** Associations between sociodemographic characteristics and high home salt consumption among the study population.

	Univariate Analysis		Multivariable Analysis	
Variable	Odds Ratio [95% CI]	*p*-Value	Odds Ratio [95% CI]	*p*-Value
**Age (ref = 18–29 years)**				
30–39 years	0.69 [0.47, 1.01]	0.06	0.99 [0.67, 1.47]	0.96
40–49 years	0.39 [0.28, 0.55]	<0.01 *	0.52 [0.36, 0.75]	<0.01 *
50–59 years	0.35 [0.24, 0.49]	<0.01 *	0.39 [0.27, 0.56]	<0.01 *
≥60 years	0.32 [0.23, 0.45]	<0.01 *	0.27 [0.19, 0.39]	<0.01 *
**Sex (ref = Male)**				
Female	0.56 [0.44, 0.71]	<0.01 *	0.57 [0.45, 0.72]	<0.01 *
**Education (ref = None)**				
Primary	1.46 [1.05, 2.01]	0.02 *		
Secondary	1.83 [1.38, 2.44]	<0.01 *		
Greater than secondary	2.84 [1.87, 4.32]	<0.01 *		
**BMI (ref = Normal [18.5–24.9 kg/m^2^])**				
Underweight (<18.5 kg/m^2^)	0.88 [0.56, 1.38]	0.58		
Overweight (25–29.9 kg/m^2^)	0.76 [0.59, 0.98]	0.04 *		
Obese (≥30.0 kg/m^2^)	0.64 [0.48, 0.84]	<0.01 *		
**Income (ref = Lower income)**				
Higher income	0.24 [0.19, 0.31]	<0.01 *	0.21 [0.16, 0.27]	<0.01 *

* Statistically significant, *p* < 0.05; BMI = body mass index, CI = confidence interval.

**Table 8:** Associations between sociodemographic characteristics and high home oil consumption among the study population.

	Univariate Analysis		Multivariable Analysis	
Variable	Odds Ratio [95% CI]	*p*-Value	Odds Ratio [95% CI]	*p*-Value
**Age (ref = 18–29 years)**				
30–39 years	0.71 [0.50, 1.02]	0.07	1.04 [0.71, 1.51]	0.85
40–49 years	0.45 [0.32, 0.63]	<0.01 *	0.62 [0.43, 0.88]	<0.01 *
50–59 years	0.41 [0.30, 0.58]	<0.01 *	0.47 [0.33, 0.67]	<0.01 *
≥60 years	0.35 [0.25, 0.48]	<0.01 *	0.29 [0.21, 0.41]	<0.01 *
**Sex (ref = Male)**				
Female	0.58 [0.47, 0.73]	<0.01 *	0.59 [0.47, 0.75]	<0.0 1 *
**Education (ref = None)**				
Primary	1.38 [1.00, 1.91]	0.05		
Secondary	1.68 [1.26, 2.23]	<0.01 *		
Greater than secondary	2.14 [1.45, 3.18]	<0.01 *		
**BMI (ref = Normal [18.5–24.9 kg/m^2^])**				
Underweight (<18.5 kg/m^2^)	1.02 [0.64, 1.63]	0.92		
Overweight (25–29.9 kg/m^2^)	0.72 [0.56, 0.92]	<0.01 *		
Obese (≥30.0 kg/m^2^)	0.67 [0.51, 0.89]	<0.01 *		
**Income (ref = Lower income)**				
Higher income	0.23 [0.18, 0.29]	<0.01 *	0.19 [0.15, 0.25]	<0.01 *

* Statistically significant, *p* <0.05; BMI = body mass index, CI = confidence interval.

## Data Availability

Data contain potentially identifying and sensitive patient information. Deidentified data used for this analysis are available upon request after signing a data access and use agreement, gaining a provision of approval by the GHESKIO ethics board, and demonstrating that the external investigative team is qualified and has documented evidence of human research protection training. Requests may be addressed to corresponding author.

## Supplementary Materials

The following supporting information can be downloaded at: https://www.mdpi.com/article/10.3390/nu14040787/s1. Table S1: Prevalence of concurrent dietary risk factors among the study population; Table S2: Self-reported fruit and vegetable consumption from the Haiti Cardiovascular Disease (CVD) Cohort study and World Health Organization STEPwise Approach to Noncommunicable Disease Surveillance (WHO STEPS) program.

## Abbreviations

BMI—body mass index; CVD—cardiovascular disease; GHESKIO—Groupe Haitien d’Etude du Sarcome de Kaposi et des Infections Opportunistes; HTG—Haitian gourde; LMIC—low- and middle-income countries; NCD—noncommunicable disease; USD—United States dollar; WHO STEPS—World Health Organization STEPwise Approach to Noncommunicable Disease Surveillance
