# Supplementary material for: Dietary Risk Factors for Cardiovascular Disease among Low-Income Haitian Adults: Findings from a Population-Based Cohort
Source: Nutrients. 2022 Feb 13;14(4):787. doi: 10.3390/nu14040787 (PMC8880283; doi:10.3390/nu14040787)
Supplement: Supplementary file 1 [file nutrients-14-00787-s001.zip › New folder/nutrients-1570553 supplementary Table S1.pdf]

Supplementary Table 1

Prevalence of concurrent dietary risk factors among the study population

Number of Risk Factors*	N (%)
0	32 (1.1%)
1	174 (5.8%)
2	259 (8.7%)
3	895 (29.9%)
4	1,275 (42.7%)
5	354 (11.8%)

* Risk factors: eating <1 serving of fruits and vegetables per day, eating fried foods ≥3 days per week, drinking sugar-sweetened beverages ≥3 days per week, high home cooking oil use, and high home salt use
