# Supplementary material for: Dietary Risk Factors for Cardiovascular Disease among Low-Income Haitian Adults: Findings from a Population-Based Cohort
Source: Nutrients. 2022 Feb 13;14(4):787. doi: 10.3390/nu14040787 (PMC8880283; doi:10.3390/nu14040787)
Supplement: Supplementary file 1 [file nutrients-14-00787-s001.zip › New folder/nutrients-1570553 supplementary Table S2.pdf]

Supplementary Table S2

Self-reported fruit and vegetable consumption from the Haiti Cardiovascular Disease (CVD) Cohort study and World Health Organization STEPwise Approach to Non-Communicable Disease Surveillance (WHO STEPS) program[1]

Country	Year	% of participants eating less than 5 servings of fruit and vegetables per day
	**CVD Cohort Study data**	
Haiti *(Port-au-Prince only)*	2019-2021	99.4%
	**WHO STEPS data – Caribbean countries**	
Aruba	2006	94.8%
Bahamas	2011-2012	90.0%
Barbados	2007	95.4%
Bermuda	2014	81.9%
British Virgin Islands	2009	92.3%
Dominica	2008	91.3%
Grenada	2010-2011	75.3%
Saint Kitts and Nevis	2007	97.3%
Saint Lucia	2012	88.3%
Saint Vincent and the Grenadines	2013-2014	94.9%
	**WHO STEPS data – Comparable GDP per capita countries[2]**	
Comoros	2011	85.7%
Ethiopia	2015	97.6%
Tanzania	2012	97.2%
Lesotho	2012	92.7%

[1] https://www.who.int/ncds/surveillance/steps/reports/en/; [2] Gross domestic product per capita (purchasing power parity adjusted) from https://en.wikipedia.org/wiki/List_of_countries_by_GDP_(PPP)_per_capita
